# Regulatory Pathways Supporting Expedited Drug Development and Approval in ICH Member Countries

**DOI:** 10.1007/s43441-022-00480-3

**Published:** 2022-12-03

**Authors:** Pedro Franco, Ritesh Jain, Elizabeth Rosenkrands-Lange, Claudia Hey, Marén U. Koban

**Affiliations:** 1 Merck Serono Limited, 5 New Square, Bedfont Lakes Business Park, Feltham, TW14 8HA Middlesex UK; 2 EMD Serono Research & Development Institute, 1299 Pennsylvania Ave., N.W., Suite 825, Washington, DC 20004 USA; 3 Merck Healthcare KGaA, Frankfurter Str. 250, F135/K04 64293 Darmstadt, Germany

**Keywords:** Expedited pathways, ICH, Development timelines, Regulatory interactions

## Abstract

Regulators and pharmaceutical companies across the world are intensifying efforts to get increasingly complex and innovative drugs to patients with high unmet medical need in the shortest possible time frame. This article reviews pathways to expedite drug development and approval available in member countries of the International Council for Harmonisation of Technical Requirements for Pharmaceuticals for Human Use and Australia. It is concluded that the increasing availability of expedited regulatory pathways and associated modernisation of regulatory systems changes the current regulatory paradigm and requires sponsors to rethink drug development and regulatory strategy. A transformation of the current sequence of regulatory submissions, favouring those countries/collaborations that are best regulatory equipped to make innovative medical need drugs available to patients in the shortest time frame is imminent.

## Introduction

Development of new drugs is a challenging and complex process associated with long development timelines and clinical trial success rates for compounds entering phase I of around 10% [1, 2]. This is despite the publication of many scientific guidelines, interactions/meetings with regulators and the establishment of dedicated review timelines by regulatory agencies in many countries. To accelerate patient access to treatment mainly in areas of serious and life-threatening diseases and unmet medical need, many regulatory authorities have put in place regulatory pathways to expedite drug development and approval. Initially, expedited pathways were introduced in the early 1990s in the United States of America (US) stimulated by the intention to allow antiretroviral treatments for patients to become available as quickly as possible to counter the threat of the AIDS pandemic [3].

Generally, the following regulatory options are available globally to expedite the development and approval of innovative drugs in areas of serious and life-threatening diseases and unmet medical need:

1. **Initial authorisation based on limited clinical data** In most countries, this pathway includes regulatory approval based on a surrogate or early endpoint. Approval through this pathway needs to be complemented by further clinical data generated post-authorisation as laid out in commitments, such as Accelerated Approval in US or Conditional Marketing Authorisation in the EU.
2. **Repeated increased interaction between the regulator and the sponsor** This option focusses on increased frequency of interactions starting early and continuing throughout drug development and involves designations as Breakthrough and Fast Track in the US or Priority Medicine (PRIME) in the EU.
3. **Shortened registration pathways **These pathways are intended to shorten the regulatory review timelines by health authorities. Regulatory agencies provide additional resources to expedite the review and evaluation of regulatory submissions. This includes pathways such as Priority Review in US or Accelerated Assessment in the EU. Other regulatory pathways that are based on reliance are not in scope of this article.

The aim of this regulatory review article is to provide an overview of the key characteristics of regulatory **expedited** pathways across members of the International Council for Harmonisation of Technical Requirements for Pharmaceuticals for Human Use (ICH). Furthermore, the aim is to discuss the potential impact on development and approval when these pathways are used. Only ICH member countries and Australia were selected since they are important markets for drug developers and the possibility to reduce drug development timelines and avoid duplication of work will have high impact. Although Mexico is ICH member since November 2021, all the regulatory pathways currently available in Mexico to expedite review are based on reliance and hence not discussed in this article.

Future regulatory perspectives, anticipating an evolving regulatory landscape aiming for expedited approvals in different regions, with potential consequences for future company submission and launch sequence strategies are also discussed. These include recent pilot collaborations such as Australia, Canada, Singapore, Switzerland and UK (Access consortium) and the US Food and Drug Administration (FDA)-led Project Orbis.

## Materials and Methods

A systematic search and in-depth analysis of information and guidelines provided by national regulatory agencies on expedited pathways was conducted, including all information available up to December 2021. In case of language barriers local regulatory experts verified guidelines content and meaning. This analysis was complemented with:

- Information from a regulatory intelligence database (Cortellis)
- Context from relevant published literature
- Information published on Health Authorities’ websites

Corresponding source documents and literature are included as references.

The authors would like to point to FRPath.org, an educational project designed to serve as the trusted repository of expertly evaluated and organized information about Facilitated Regulatory Pathways, although this source was not used for the purpose of this paper.

## Results

### Country-Specific Information on Available Expedited Pathways

The most recent information and procedure details collected on regulatory pathways in different ICH members is summarised in the Tables 1, 2 and 3 (countries listed in alphabetical order). The tables classify the regulatory pathways according to the criteria “Initial authorisation based on limited clinical data” (Table 1); “ Repeated, increased agency interaction” (Table 2) and ‘Shortened registration pathways ‘ (Table 3).

**Table 1:** Overview of pathways for authorization based on limited clinical data

Country	Procedure name	Available since	Eligibility	Scope	Characteristics and Data package	Timelines	References
Australia	Provisional approval based on early evidence data package	2018	• Life-threatening or seriously debilitating condition; • No approved treatment available or likelihood of significant improvement efficacy/safety based on preliminary clinical data; • Major therapeutic advantage over existing treatment or standard of care is likely	New prescription medicine or new indication	One application per indication Application includes: • Comprehensive non-clinical data on safety, quality and compliance with Good Manufacturing Practice; • Justification of any early or surrogate endpoints used; • Preliminary clinical data; • Comparison against other approved drugs; • Demonstration of major therapeutic advance over Standard of Care (SoC); • Robust development plan showing that comprehensive clinical data on safety/efficacy is submitted within 6 years of registration Rolling submission of clinical data (pre-agreed) is accepted Provisional registration valid for 2 years If comprehensive confirmative data cannot be collected (e.g., orphan designation) the standard regulatory pathway is likely to be applicable	Pre-submission meeting 3 months prior to request Timing of request: 2–3 months prior to submission. Provisional determination in 20 working days and valid for 6 months (extension possible) Review time: 220 working days from start of first assessment	[15, 28]
Brazil	Special registration pathway for orphan drugs	2017	• Serious debilitating condition affecting not more than 65 in 100,000 people in Brazil; • New drug, which was never commercialized in Brazil to treat, diagnose or prevent the rare disease	Marketing authorization application, Clinical trial application, GMP certification	Assessment based on phase 2 data of finished studies and studies in progress (phase 3 may or may not be ongoing) Bibliographic references, in vitro/in vivo comparability studies, BA/BE studies with international comparator to complement the clinical package Pre-submission meeting (compulsory) to be requested within 60 days after international submission Meeting takes place within 60 days after request Application to be submitted within 30 days after pre-submission meeting	First review round: 30 days Clock stop: maximally 30 days Review of responses: 45 days	[29]
Canada	Notice of Compliance with Conditions (NOC/c)	2016	• Serious life-threatening or severely debilitating disease or condition: • No drug is presently marketed in Canada; • Or expected significant improvement in Benefit/Risk (B/R) over existing products	New drug submission (NDS), supplemental new drug submission, abbreviated new drug submission or supplement to abbreviated new drug submission	Based on promising evidence Prior to early access and approval, the sponsor commits in writing to design, carry-out and report on confirmatory trials to verify the clinical benefit of the drug	Timing of request: ≤ 60 days following notification of eligibility Review target is 200 working days but time will depend on complexity of submission as amount of data in NOC/c dossier is usually equivalent to standard NDS	[15, 30]
China	Conditional approval procedure	2020	• Medicines used for serious, life-threatening diseases for which no effective treatment is available and there is an urgent medical need or for rare diseases; • Vaccines needed to respond to major public health emergencies that are urgently needed as defined by the National Health Commission	Initial applications, new indications	Timing of request: during development. Application for conditional approval is to be discussed with centre for drug evaluation (CDE) before submission and available data (clinical, pharmaceutical, pharmacological and toxicological) and proposed post-marketing clinical trial protocol and timeline for completion should be shared with CDE Application for marketing application can be made supported by interim analyses but applicant commits to complete the clinical package and post-approval obligations		[31, 32]
European Union	Conditional Marketing Authorisation	2004	Products intended for treatment, prevention or medical diagnosis of seriously debilitating or life-threatening diseases Medicines with Orphan Drug Designation (to be requested, not automatic)	Initial marketing authorization application (MAA)	Authorization is based on early evidence data package provided that: • The B/R balance is favourable; • Unmet medical needs are met; • The applicant will be able to provide comprehensive data; • Benefit of immediate availability on the market outweighs the risk inherent to less than comprehensive data package Authorization valid for 1 year and renewable Switched to regular MAA once comprehensive data have been provided and specific obligations fulfilled	Timing of request: 6–7 months before submission	[15, 33, 34]
European Union	Approval under exceptional circumstances	2004	It is not possible to generate comprehensive data on efficacy/safety under normal conditions of use because of: • Indication too rare; • Not possible in present state of scientific knowledge; • Collecting information is against accepted ethical principles	Initial MAA	Authorisation is based on less than comprehensive data • Request includes a statement on appropriateness of granting MAA under exceptional circumstances Dossier includes: • Listing of data that cannot be comprehensively provided; • Justifications on grounds for approval; • Proposal for specific obligations to ensure risk management Authorisation valid for 1 year and reviewed annually to re-assess the B/R balance No obligation to supply a comprehensive data package after approval	Timing of request: at the time of intention to submit MAA (> 6 months before submission)	[35–37]
Japan	Conditional early approval	2017 (officially legalised in 2019)	Drugs targeting particularly high medical needs in serious target disease with limited treatment options where it is difficult to conduct confirmatory clinical trials due to small number of patients or long trial periods	Initial MAA, new indications/major advances	Eligibility for conditional early approval to be discussed in early stage (prior to late phase 2 clinical studies) in priority face to face interviews. MAA can be submitted based on exploratory non-confirmatory clinical trials (e.g., surrogate or interim data, oversees only data) together with other evidence. There should be proof of a certain degree of efficacy and safety Full approval is granted but post-approval conditions to reconfirm efficacy/safety will be agreed and will be evaluated promptly	Priority review is foreseen in this procedure (see Table 3) Timing of request: during development	[15, 38, 39]
Republic of Korea	Conditional approval with Phase 2 results	2021	Oncology products, orphan drugs, cell therapy for life-threatening or irreversible disease that use surrogate endpoints	Initial MAA	Ability to base studies on phase 2 studies with surrogate endpoint		[40]
Republic of Korea	Full Approval with Phase 2 Results	2021	Unmet need and less than 2000 cases in a year		Ability to base studies on phase 2 studies with surrogate endpoint		[40]
Singapore	No official procedure/guidance, case-by-case only		Products addressing unmet medical need conditions and granted priority review	Initial MAA	Granting of the initial marketing authorization based on a limited clinical data set is feasible. Potential post-approval commitments will need to be fulfilled. It seems that Singapore is accepting reduced clinical packages in the context of project Orbis and Access		
Switzerland	Temporary authorization	2019	• Medicine used to diagnose, prevent or treat disease that can lead to serious invalidity, severe suffering possibly resulting in death or death of the patient in a short term; • No alternative equivalent medicinal product authorized in Switzerland; • Major therapeutic benefit expected; • Comprehensive data can be provided before the temporary authorization expires; • Generating and assessing comprehensive data takes too long, resulting in irreversible damage to patients	Initial MAA	Eligibility to be requested in advance through an accelerated application hearing 2–12 months before submitting the application. Request granted/denied within 30 days. Dossier to be submitted within 6 months after official decision Dossier contains full quality and non-clinical information and promising clinical data Comprehensive data to be generated after approval according to an agreed time schedule Authorization is valid for 2 years, which can be extended upon request in scientifically justified exceptional cases When all conditions are fulfilled, the temporary authorization will be changed to a regular authorization valid for 5 years	Standard regulatory review: MAA 330 days Temporary authorization review: 140 calendar days (5 days validation, 1st round 65 days, 2nd round 50 days, labeling 20 days—excl. clock-stops) Switch to ordinary approval of temporary authorization: 330 calendar days (reduced if medicine is an approved orphan medicine in EU/US or intended to prevent transmissible infections and FDA/EMA reviews are available)	[41, 42]
Taiwan	Accelerated approval	2013	Medicine addresses unmet medical need and provides major clinical advance Indication for 1 of following: • Serious disease; • Orphan status in a reference country; • Hard to acquire or manufacture Clinical trials employing surrogate endpoints are able to predict clinical endpoints	New chemical entities, new combination, new indication, new route of administration	The Health Authority is willing to grant conditional approval based on an effect on a surrogate endpoint and phase 2 data. This requires completion of post-marketing confirmatory trials to prove clinical efficacy. If these cannot be provided, the license will be withdrawn	Timing of request: 2–3 months prior to submission	[43]
United Kingdom	Conditional Marketing Authorisation (CMA)	2021	• Products intended for treatment, prevention or medical diagnosis of seriously debilitating or life-threatening diseases • Medicines with Orphan Drug Designation (to be requested, not automatic—same as EU)	Initial MAA	There is no specific application route for a CMA; applicants should submit their MAA dossier as for a full Marketing Authorisation Include justification for a CMA and indicate clearly what clinical studies are underway and when comprehensive clinical data will become available Eligibility for a CMA will be determined by the MHRA at the time of MAA assessment CMAs will be valid for one year and will be renewable annually	As for standard MAA	[44]
United Kingdom	MAA under exceptional circumstances	2021	Medicines where a comprehensive data package cannot be provided, because the condition to be treated is rare or because collection of full information is not possible or is unethical. (same as for EU)		Applicant must show that the applicant is unable to provide comprehensive data on the efficacy and safety of the medicinal product under normal conditions of use Specific obligations are likely to be imposed and will be communicated to the applicant during review Conditions and compliance will be reviewed at least annually		[45, 46]
United States of America	Accelerated approval program Note: failure to demonstrate clinical benefit can result in withdrawal from the market. If clinical benefit trial is successful regular approval is granted	1992	• Medicines intended for serious and life-threatening disease; • Meaningful benefit over available therapies	Initial NDA/BLA and supplemental NDA/BLA	Possibility of accelerated approval to be discussed with FDA during development. Approval initially based on surrogate or intermediate clinical endpoints that are reasonably likely to predict a clinical benefit (i.e., not a validated clinical endpoint) For therapies approved under this pathway it is required to demonstrate clinical benefit in post-marketing studies	Timing of request: during development	[15, 47]

**Table 2:** Overview of pathways involving repeated, increased interaction between the regulator and the sponsor

Country	Procedure name	Available since	Eligibility	Scope	Characteristics and Data requirements	When and how to apply/timelines	References
Australia	Not applicable						
Brazil	ANVISA advice on innovative approaches/new science	2017	Medicines to treat rare diseases	Initial MAA, post-authorisation (new therapeutic indication, generic drug to avoid shortages in the market, vaccines), prior consent in clinical research of drugs	Provides advice on innovative approaches or new science prior to submission		[29]
Canada	Not applicable						
China	Breakthrough designation (BTD)	2020	Innovative or modified new medicine for prevention and treatment of: • Serious debilitating disease; • Disease associated with irreversible morbidity or high mortality No other treatment or prophylaxis or clinical superiority over standard of care in China Not open for products approved outside China Note: Drug will be removed from program in case of safety issues or lack of superiority	Initial MAA or new indication	The scheme foresees additional interactions/meetings during drug development at key stages with prioritized resource allocation. BTD gives high possibility of priority review (see Table 3) and rolling submission Prioritizes on-site inspections and QC testing	Application to be made for each eligible indication separately. To be requested during clinical trials (before start phase 3). Application procedure 45 + 5 days. Within 6 months after inclusion in the program, applicant can submit application for first communication in accordance with category I meeting	[48]
European Union^a^)	Priority Medicines (PRIME)	2016	Medicines that may offer a major therapeutic advantage over existing treatments, or benefit patients without treatment options	Initial MAA	Close interaction at key development milestones providing support to optimize generation of robust data Early appointment of rapporteur Potential for accelerated assessment (Table 3) and conditional marketing authorization	To be requested during exploratory clinical development based on availability of preliminary clinical evidence in patients substantiating that the product may offer major therapeutic advantage over existing treatments or benefit patients without treatment options. Designation in 40 days	[49]
Japan	Pioneering drug designation (formerly known as Sakigake)	2015	• Innovative medicine (e.g., first in class); • Serious target disease (life-threatening, high impact on daily life; • Outstanding efficacy/safety; • Early development in Japan and at least simultaneous MAA with other countries	Initial MAA	Offers priority consultation (one month consultation compared to 2 months in regular process), prior assessment, built-in rolling review, priority review (see Table 3) Request for designation to be substantiated with clinical data Of note, in case of developments requiring companion diagnostics these will have to be reviewed (by PMDA) in parallel with the medicine	Granted twice a year (April and October) Granted in approx. 60 days	[50, 51]
Republic of Korea	Not applicable						
Singapore	Not applicable, regular pre-submission consultation						
Switzerland	Not applicable, regular pre-submission consultation, scientific advice and clarification meetings						
Taiwan	Breakthrough Therapy	2019	All criteria must be met • New medicines and indications in the areas of unmet medical need; • Serious or rare target disease; • At least one clinical trial in Taiwan, especially clinical trials in the early phase	Initial MAA and new indications	Characterized by early interactions with the Health Authority, including intensive guidance during drug development (every 3 months), possibility to apply for Module-based rolling review and eligibility for priority review (Table 3) Application based on early clinical evidence for substantial improvement over existing therapies	To be applied for before the end of phase 2 study final report. Designation in 1–2 months	[43]
United States of America	Breakthrough Therapy Designation	2012	Serious condition and possibility to demonstrate substantial improvement over available therapies	Initial NDA/BLA and new indications for approved products	Access to FDA’s intensive guidance on efficient drug development from Phase 1 onward FDA commitment to involve senior managers Access to rolling review (see Table 3) Potential for priority review (see Table 3) Application based on preliminary clinical evidence indicative of potential substantial improvement on clinically significant endpoint over available therapies	Request should be made with IND or after; ideally, no later than the end-of-phase 2 meeting FDA aims to respond within 60 calendar days Designation can be lost if data no longer support eligibility	[47]
United Kingdom	ILAP (Innovative Licensing and Access Pathway)	2021	• Life-threatening or seriously debilitating condition or significant patient or public health need; • Innovative medicine or medicine for rare disease/special populations or development aligning with UK public health priorities; • Medicine has potential to offer benefits to patients	Initial MAA (multiple indications) and clinically significant new indication for already approved medicine	Early stage discussions involving also National Institute for Health and Care Excellence and Scottish Medicines Consortium	First step is Innovation Password application. Within 4–6 weeks after receipt of application form, meet with MHRA to discuss how eligibility criteria are fulfilled. Designation granted in 8–10 weeks (4 weeks after meeting) Multiple entry points depending on stage of development as early as availability of non-clinical data	[52]
United States of America	Regenerative Medicine Advance Therapy (RMAT) Program	2016	Regenerative therapies (cell therapy, therapeutic tissue engineering products, human cell and tissue products or combinations of these) intended to treat, modify, reverse or cure a serious or life-threatening disease or condition	Initial MAA	Offers all breakthrough therapy designation features (see above) including early interactions to discuss surrogate or intermediate endpoints	To be requested preferably at the time of IND but no later than end of phase 2 meeting	[53]

^a^Until 2018 EMA ran a pilot scheme under the name Adaptive Pathway for approval in stages (e.g., starting with a restricted patient population, then expanding to wider patient populations as more data are generated). For this pathway existing tools such as conditional marketing authorization and scientific advice were used [54]

Table 1 illustrates that authorisation based on limited clinical data is a regulatory option in all ICH members but differences in eligibility and scope exist. In the case of Singapore, no specific guideline is available but during the COVID-19 pandemic, remdesivir was conditionally authorised demonstrating that in emergency situations the possibility exists.

Repeated, increased interaction between the regulator and the sponsor is a more recent regulatory tool to expedite drug development. Thus far, Brazil, China Breakthrough Designation (BTD), US FDA (BTD and Regenerative Medicine Advance Therapy (RMAT) program), EU (PRIME), Japan (Sakigake), Korea and Taiwan offer this option of repeated, close engagement with the Health Authority during drug development. In many cases, drugs that qualify for an increased interaction pathway are also eligible for one or more of the other expedited pathways (Table 2).

As summarized in Table 3, regulators in all ICH members offer shortened marketing authorisation application review pathways. Eligibility requirements and scope differ across ICH members. Shorter pathways are mostly achieved through repeated, intensified development and pre-submission interactions, expedited review or rolling or split submissions or a combination of these.

**Table 3:** Overview of shortened registration pathways *(excluding reliance)*

Country	Procedure name	Available since	Eligibility	Scope	Characteristics	Review timelines	References
Australia^a,b^	Priority review	2017	Criteria: • Serious condition; • Comparison against registered therapeutic goods (medicines with provisional registration are excluded from comparison); • Major therapeutic advance	New prescription medicine or new indication based on full dossier (i.e., provisional approved medicines are excluded)	Timing of request: 3 months prior to submission of an application for marketing authorization (MAA). 20 days for decision (excluding response time to information requests). Optional pre-submission meeting 2 months before request If eligible Pre-submission Planning form to be completed before submitting (max. 6 months)	Standard regulatory review: 255 working days Priority review: 150 working days Median time to approval (NAS) 2021: Expedited: 221 days Standard: 354 days	[15, 55, 56]
Brazil^a^	Priority review resolution	2018	One of the following criteria: • New or innovative products; • Significant improvement in emergent/neglected/rare diseases, vaccines for national immunization programme, public health emergencies, first generic	New applications for marketing authorization, post-approval changes (new indications, generic applications in the context of shortages) and clinical trial applications	Timing of request: at the time of the marketing authorization application and 60 days after 1st international submission Procedure paused (clock stop) in case of questions Conditions: • Medicine must be marketed within 365 days after register approval; • New chemical entities must submit price request within 30 days after register approval	Standard regulatory review (calendar days): 365 for MAA and 180 for post-marketing changes Priority review (calendar days): 120 for MAA, 60 for post-marketing changes, 45 for clinical trials ANVISA can request extension of 1/3 of each period Median time to approval (NAS) 2021: no data available	[57]
Canada^a,b^	Priority review	2005	Medicine for a serious life-threatening or severely debilitating disease or condition: • No alternative therapy is currently marketed or, • Expected improvement of overall Benefit/Risk compared to existing therapies/preventatives or diagnostic agents for disease/condition that is not adequately managed by a drug marketed in Canada	New Drug Submission or supplemental New Drug Submission	Timing of request: Written request in advance of the filing of the drug submission to the Director of the appropriate bureau at Health Canada NDS to be submitted within 60 calendar days but not prior to the date of issuance of the acceptance letter Data include substantial evidence of clinical effectiveness	Standard Regulatory Review: 450 days Priority Review target: 180 calendar days Median time to approval (NAS) 2021: Expedited: 207 days Standard: 343 days	[15, 58, 59]
China	Priority review	2020	Intended for: • Medicines in the area of unmet medical need • Shortages of drugs with urgent clinical need • Innovative drugs • Improved new drugs for major infections and rare diseases • Paediatric medicines • Medicines eligible for conditional approval (see Table 1) and Breakthrough therapy (see Table 2) • Other circumstances considered by National Medical Products Agency	New applications or new indications	Timing of request: prior to submission. Before submitting an application for priority review, the applicant must contact the centre for drug evaluation to discuss whether the existing clinical data meet the requirements of the eligibility criteria. If eligible application for priority review to be submitted with supporting materials at the time of MAA	Standard Regulatory review: 16–20 months Priority review standard: 11–13 months (130 working days) Priority review for orphan drugs authorized overseas but not marketed in China: 70 working days Median time to approval 2021: no data available Priority review will switch to standard review if conditions are no longer met	[31]
China	Special review and approval procedure	2020	Public Health emergencies, threats	New applications and new indications	Characterised by regulatory flexibility and short evaluation timelines. Use of the medicine may be limited to the emergency period only	Standard regulatory review Special review and approval	[31]
European Union	Accelerated assessment	2004	Major interest to public health: • Unmet medical need or • Major therapeutic advantage/innovation	New applications	The intent to request is to be discussed in advance. Justification is needed that early access of the product is of major interest to public health	Pre-submission meeting highly recommended (6–7 months before MAA). Requests should be submitted at least 2–3 months before submitting the MAA Standard regulatory review: 210 days (excl. clock-stop) Accelerated assessment: 150 days (excl. clock-stop) Median time to approval (NAS) 2021: Expedited: 250 days Standard: 434 days Can revert to regular timeline if it is deemed no longer appropriate to conduct review under accelerated conditions	[15, 60, 61]
Japan	Priority Review	2020	• Orphan drug • Drugs designated for pioneering drugs (Sakigake) review (see Table 2) • Pioneering drugs, devices or regenerative drugs (regular or special purpose) • New drugs, devices or regenerative drugs indicated for a serious disease or with efficacy/safety clearly medically superior to existing treatment modalities	New applications or new indications/major advances	Timing of request: prior to submission. Request for priority review to be indicated in the application form, including reason for eligibility. If available, evaluation report based on consultation on applicability drug priority review products to be attached. PMDA compiles opinion and MHLW will decide. Decision announced at the time of approval	Standard regulatory review: 12 months Priority review including orphan drugs: 9 months,Pioneering Drugs (Sakigake) 6 months. Median time to approval (NAS) 2021: Expedited: 266 days Standard: 331 days	[15, 38, 62]
Republic of Korea	Priority Review	2021	Medicines for: • Life-threatening or critical diseases with no existing treatment or offering significant efficacy improvement • Public health crisis		Timing of request: Prior to submission Reviewed in 30 working days. Outcome is announced on the agency webpage	Standard regulatory review: 120 days when DMF review is included, 90 days when not Priority review reduces timeline by 75% compared to standard Median time to approval (NAS) 2021: no data available	[63]
Republic of Korea	Rolling submission	2021	New biotech like Cell therapy and Gene therapy and biosimilars	Expedited submission	Standard regulatory review: 120 days when DMF review is included, 90 days when not		[40, 63–67]
Singapore^a,b^	Shortened registration procedure—priority review	2017	• Only applicable to products eligible for abridged evaluation pathway and that hence have already been evaluated and approved by at least one drug regulatory authority (any) • Product for a life-saving disease where there are unmet medical needs • Product demonstrates potential to address the unmet medical need (lack of alternative treatment, significant improvement in efficacy/safety) • Disease conditions that are of local public health concern will be given primary consideration	Initial MAA	To be requested at the time of application for Marketing authorisation Justification to be provided in Module 1 To be granted/denied at the time of acceptance of the MAA application	Standard regulatory review: 270 working days (excl. clock-stops) Abridged evaluation: 180 working days (excl. clock stops) No dedicated official timelines for priority review available but expected to be shorter than abridged pathway timelines. Median time to approval 2021: no data available	[68]
Switzerland^a,b^	Fast-track procedure (FTP)	Initial Introduction in 1997	• Promising prevention against, treatment for a severe disabling or life-threatening disease • Currently available treatment unavailable or unsatisfactory • Expected high therapeutic benefit • Clinically relevant trial	Initial MAA or variations	Request to be submitted as part of an Accelerated Application Hearing between 2–12 months prior to the planned submission. Decision will be made within 30 days Dossier may be submitted 2 months after granting of FTP	Standard regulatory review: 330 calendar days, indication 270 calendar days FTP review: 140 calendar days (5 days validation, 1st round 65 days, 2nd round 50 days, labelling 20 days—excl. clock-stops) Median time to approval (NAS) 2021: Expedited: 245 days Standard: 399 days	[15, 69]
Switzerland^a,b^	Procedure with prior notification (PPN)	2013	Any new active substance or approved medicine	Initial MAA or indication extension	Special service offered at twice the normal fee	Standard regulatory review: 330 calendar days, indication 270 calendar days PPN review new MAA: 264 calendar days PPN review extension indication: 216 calendar days Median time to approval (NAS) 2021: Expedited: 245 days Standard: 399 days	[15, 70]
Taiwan	Priority review	2019	Medicine addresses unmet medical need and provides major clinical advance	New chemical entities, new combination, new indication, new route of administration	Timing at request: 2–3 months prior to submission. Same standards for dossier Unmet medical need should be justified unless the drug or indication is under a special national scientific research and development program	Standard regulatory review: 360 days (15–18 months) Priority review: 240 days (10–12 months) Median time to approval (NAS) 2021: no data available	[43]
Taiwan	Abbreviated review Category I	2010	New chemical entities already approved by 2 of the 3 regulatory agencies (FDA-US, EC/EMA or MHLW/PMDA) with bridging study waiver	Initial MAA	Dossier to include the full assessment reports issued by the reference health authorities, the Risk Management Plan and the post-marketing commitment reports from the reference health authorities. BSE waiver (lead-time 6–9 months) and designation (lead time 1 month) need to be available before submission of the MAA	Standard regulatory review: 360 days Streamlined review: 180 days Median time to approval (NAS) 2021: no data available	[43]
United Kingdom^a,b^	Rolling review	2021	All new active substances and biosimilars	Initial MAA	MAA is submitted in separate modules for starting a pre-assessment phase	Median time to approval (NAS) 2021: no data available	[71]
United States of America^a^	Priority review program	1992	Drug products intended for a serious condition, expected to provide a significant improvement of efficacy/safety of the treatment, diagnosis or prevention	Initial NDA/BLA or efficacy supplement	Timing of request: at time of marketing authorization application FDA will inform the applicant within 60 days in case they decide to grant priority review	Standard regulatory review: 12 months for New molecular entity (NME) NDA and original BLA. 10 months for efficacy supplements Priority review: 8 months for a NME NDA and original BLA and. 6 months for an efficacy supplements Median time to approval (NAS) 2021: Expedited: 242 days Standard: 365 days	[15, 47]
United States of America^a^	Fast track Program	1997	Drug products intended for treatment of serious or life-threatening disease or a condition with an unmet medical need		May be requested at the time of an IND or thereafter, ideally no later than pre-submission meeting. FDA accepts/denies within 60 calendar days of receiving the request Provides opportunity for: • Frequent interactions with FDA • Rolling review—requires submission of complete CTD modules • Potential for priority review Applicant must demonstrate that drug meets eligibility criteria and has potential to address unmet need based on non-clinical and early clinical data	Note: Fast track designation can be withdrawn if data no longer support drug potential	[47]
United States of America^a^	Split Real Time Application Review (STAR)	2022	Applies to all therapeutic areas	Efficacy supplements for novel uses of existing therapies for patients with a serious condition with unmet medical needs	Part 1 can be submitted as early as 2 months before completing the application. Sponsor submits the efficacy supplement minus the final clinical study reports and clinical summaries	Although the review clock begins with submission of Part 2, FDA will set an action date to be at least 1 month earlier than the normal 6-month goal date for a priority review application	[72]

^a^Countries participating in ORBIS: Participation can potentially shorten overall review time by collaborative review

^b^Countries participating in ACCESS consortium. Participation in the ACCESS consortium can potentially shorten overall review time by work-sharing

### Additional Regulatory Collaborations—Perspectives

To further enhance the efficiency of national regulatory systems, regulatory authorities are also looking for alignment and creating synergy, with the aim to reduce duplication and bring innovative drugs to patients in their countries as early as possible. Several initiatives such as Project Orbis, Access consortium, and the ASEAN joint assessment have been started that seek to share review experience and burden and to foster close exchange between participating regulators, preferably throughout the life cycle of a drug (Table 4).

**Table 4:** Summary characteristics of the joint assessment collaborations ORBIS, ACCESS and ASEAN JACG

Characteristic	ORBIS	ACCESS	ASEAN JACG
Therapeutic areas	Oncology only; NME or efficacy supplement (indication extension)	Across indications; NME or efficacy supplements (indication extension), new active substances, generics and biosimilar drugs	Across indications
Application process	By invitation from FDA (however, may be requested by sponsors)	By application via expression of interest form 3–6 months prior to submission	Selection by NRA supported by list of priority pharmaceutical products
Participating countries	US, Canada, Australia, Brazil, Israel, Singapore, Switzerland, UK, others to be determined Currently out of scope: EU, China Participating agencies have to agree on participation based on capacities prior submission	Australia, Canada, Singapore, Switzerland, UK (must include at least 2 of these countries)	ASEAN NRAs: Brunei, Cambodia, Indonesia, Laos, Malaysia, Myanmar, the Philippines, Singapore, Thailand, and Vietnam (at least three must participate)
Dossier	Country-specific—similar in content, but each application should meet the local content requirements	Required to be overall the same dossier, local differences need to be highlighted	Overall same dossier
Time window for submission in participating countries	Type A Orbis: “Simultaneous” Type B Modified Orbis: Within 3 months of FDA filing date Type C Written Report Only: post FDA regulatory action	Within 2 weeks	Simultaneous submission
Regulatory pathways	Can be different (standard, priority, etc.—pathways can be mixed)	Same pathway required: either standard OR priority	Same pathway
Review process	Parallel review of dossiers, frequent telecons of participating agencies	Work-sharing assignment of lead agency per Module (M3-5); peer review and national phase	Parallel review. Assignment of lead agency to coordinate and facilitate assessment, liaise with sponsor and facilitate support from well-sourced NRA
Questions	Ideally consolidated, but not always the case	Consolidated, batched or rolling questions (agreed upfront), but extra national questions; fixed milestones	Consolidated
Decision making	Independent	Independent	Independent
Labelling decision	Independent	Independent	Independent (not specified as such)

NME = New Medical Entity; US = United States; FDA = Food and Drug Administration; UK = United Kingdom; EU = European Union; NRA = National Regulatory Agency

#### Project Orbis

Project Orbis, an initiative from FDA’s Oncology Center of Excellence provides a framework for concurrent submission and review of oncology drugs among partner international regulatory agencies [4]. This initiative was launched in 2019 with three participating agencies (FDA, the Australian Therapeutic Goods Administration (TGA) and Health Canada); however, new Participating Orbis Partners (POP) were recently added such as Singapore’s Health Sciences Authority (HSA), Switzerland’s SwissMedic, Brazil’s ANVISA, UK’s Medicines & Healthcare Products Regulatory Agency (MHRA) and Israel’s Ministry of Health (MoH) [5–9].

Submitted dossiers are similar in content but must meet national regulatory requirements of each country. The review process is based on collaborative review (involving FDA as primary coordinator plus at least one of the POPs) but each of the POPs performs its own review and remains fully independent in making its regulatory decision. Mutual confidentiality agreements have been set up to allow to share assessment information among partners. Cross-functional assessments are facilitated through an Assessment Aid (FDA template) and questions can be coordinated by FDA (but each POP can send questions).

In the first year of the project (June 2019–June 2020) a total of 60 oncology marketing applications were received. The first New Medical Entity (NME) under Orbis was approved on April 17, 2020. The median time gap in the submission date to the FDA and to the POP was 0.6 months (range −0.8 to 9.0 months). The median time to approval was similar between FDA (4.2 months, range 0.9–6.9 months, *N* = 18)) and the POP (4.4 months, range 1.7–6.8, *N* = 20) [10].

#### Access Consortium

Initiated in 2007, the Access (formerly ACSS) Consortium is a coalition of ‘like-minded’, medium-sized regulatory authorities (currently Australian TGA, Health Canada, SwissMedic, HSA Singapore and UK MHRA) that work together to promote greater regulatory collaboration and alignment of regulatory requirements [11]. It also aims to bring drugs faster to patients. Access offers a work sharing pilot for coordinated assessment of new applications or indication extensions filed in at least two Access countries. A single assessment of a common dossier (Module 2–5) facilitated via work-sharing between participating members is followed by a national step where each jurisdiction takes separate sovereign approval and labelling decisions. All participating countries need to agree on either standard or priority review. In contrast to project Orbis, Access review can be requested for drugs for any therapeutic area. Since the first simultaneously reviewed Access submission in 2017 until July 2021, 12 drug submissions have been evaluated; 16 drugs are under active review; and an additional seven applications are in pre-filing planning [12].

#### ASEAN Joint Assessment

The Association of Southeast Asian Nations (ASEAN) is a collaboration of 10 member states aiming to accelerate economic growth, social progress and cultural development. The collaboration also includes harmonisation of regulatory requirements for pharmaceutical drugs. Participation is open to all ASEAN National Regulatory Agencies (NRA) on a voluntary basis and is specific for the drug being assessed. The Joint Assessment Coordinating Group (JACG) will assist NRAs to define drugs eligible for the joint assessment procedure, supported by a list of priority pharmaceutical drugs which will be periodically reviewed and published. Joint assessment will be implemented when a minimum of three NRAs decide to participate. For each joint assessment, a lead NRA will be appointed who coordinates and facilitates the assessment work. The final decision on the application will be made through the regular decision-making process of each participating country. A stable ASEAN-wide IT platform is being developed to support the operation of the collaborative processes (dossier submission, online review, consolidated list of questions, online responses from sponsors and joint assessment reports). The JACG has completed two joint assessments (both concerning antimalarial drugs). In one of the examples, all steps of the process up to release of the final joint assessment report were completed in less than 5 months [13].

EMA Pilot Project OPEN—most recently, triggered by the COVID pandemic, the European Medicines Agency (EMA) launched in December 2020 the OPEN pilot to increase international collaboration on the evaluation of COVID-19 vaccines and therapeutics. The objective is to share scientific expertise, while each agency remains fully independent. Participating agencies are the TGA, Health Canada, the Japanese Pharmaceuticals and Medical Devices Agency (PMDA), SwissMedic and the World Health Organization (WHO). EMA will evaluate the pilot and has announced publicly to expand the OPEN scheme to antimicrobial resistance; quality topics / variations, products going through the PRIME scheme and medicines combating health threats or public health emergencies [14].

## Discussion

In an effort to improve factors that impact timelines for drug development and approval, all ICH members have established pathways with expedited review timelines and initial approval based on limited clinical data as part of their regulatory toolkit. Pathways offering repeated, increased interactions with regulators to expedite drug development are established by many but not all ICH members. A possible explanation may be that these interactions are resource intense. The FDA has the longest experience with expedited pathways. Both priority review and accelerated approval were introduced in 1992. Other jurisdictions are rapidly catching up and have initiated pathways that shorten review timeline (in particular China, but also Brazil for rare diseases, Taiwan, Republic of Korea). ICH membership is expected to have a positive impact on this process and the growing scientific expertise may increase further availability of increased interaction pathways for the often more complex high medical need drugs. Similarly, by being a new ICH member, it is expected that Mexico will modernise its regulatory environment in the near future.

The FDA’s Breakthrough Therapy Designation and Fast Track designation have been well established as development regulatory tools for over a decade and have contributed significantly to expedite drug development. BTD is one of the most sought designations for drugs for serious conditions where preliminary clinical evidence indicates that the drug may demonstrate substantial improvement over available therapy on a clinically significant endpoint(s). Other ICH members have started to offer repeated, increased interaction between the agency and the sponsor during development. Examples are Japan (pioneering drug designation previously referred to as SAKIGAKE) and the EU (PRIME—PRIority MEdicines), but also several of the emerging markets such as China (Breakthrough designation) and Taiwan (Breakthrough Therapy). The UK, now independent from the EU, has introduced ILAP—Innovation Licensing and Access Pathways, which not only involves the regulators but also important payers (National Institute for Health and Care Excellence and Scottish Medicines Consortium). When pursuing respective designations sponsors must align them with the subsequent filing strategy/sequence: for example, upholding of a Sakigake designation requires the first global filing to be in Japan or simultaneous in another first country.

### Utilization of Expedited Pathways Across ICH Members

The extent to which the expedited pathways are being used and the impact on the median approval times have been analysed for a number of jurisdictions as part of the Centre for Innovation and Regulatory Science (CIRS) annual analysis of New drug approvals across indications by six major regulatory agencies (EMA, FDA, PMDA, Health Canada, SwissMedic and TGA) [15].

The CIRS data show that the use of expedited review versus standard review pathway differs greatly between jurisdictions. It is highest in the US where 71% of New Drug Approvals were reviewed under the expedited pathway (Priority review) in 2020, followed by Japan (45%), Canada (26%) and Australia (14%). The EU and Switzerland have the lowest percentage of expedited review (Accelerated Assessment) of completed new marketing authorisation applications (9% and 8% respectively). For the EU this might be impacted by the fact that drugs that are eligible for accelerated assessment often (50–60%) revert to the standard timelines, in case major objections are raised in the course of the assessment [16]. Between 2016 and 2020, over half (35 of 67) of Accelerated Assessment requests for non-PRIME designated drugs were rejected as compared to about 15% (3 out of 22) for PRIME designated drugs [16, 17]. Based on these figures, the ongoing revision of the EU pharmaceutical legislation provides an excellent opportunity to revisit EU’s expedited tools and how they are combined to gain competitive advantage when compared to other regulatory authorities (e.g., FDA; PMDA).

When focussing on review time gain (Table 5), a rather consistent gain of around 40% emerges between the ICH members for expedited versus standard marketing authorisation application review.

**Table 5:** Time gain when using expedited versus standard review pathways for new active substance approvals, complemented and adapted from CIRS [15]

Jurisdiction	Median Approval Time^a^ in days (based on overall approval time)			Percentage time saved with expedited review^b^
	Expedited	Standard	Benefit (reduction in days)	
Australia (TGA)	221	354	133	38%
Canada (Health Canada)	207	343	136	40%
EU (EMA/CHMP)	250	434	184	42%
Japan (PMDA)	266	331	108	43%
Switzerland (SwissMedic)	245	399	154	33%
US (FDA)	242	365	123	34%

EMA = European Medicines Agency; FDA = US Food and Drug Administration; PMDA = Japan Pharmaceuticals and Medical Devices Agency, TGA = Australian Therapeutic Goods Administration

^a^Calculated from day of submission to date of approval by agency. Includes EU Commission time for EU

^b^Calculated as reduction in days/standard review time in days × 100%

A CIRS survey in 2019 showed that both FDA’s BTD and PMDA’s pioneering drug designation (Sakigake) had a positive qualitative impact both within companies and outside (patients, physicians, regulators and investors) [18]. FDA fast track and EMA PRIME were less positively valued in this qualitative survey. PRIME is less valued due to the short time window of application and lack of availability for new indications. The drawbacks are recognised in EMA’s 5-year analysis of the PRIME scheme [16]. Between 1 January 2018 and 30 June 2021 18 drugs that previously received the PRIME designation have been authorised (10 of them received a conditional Marketing Authorisation). Overall, 25% of PRIME eligibility applications are accepted, the therapeutic area of haematology was highlighted as having a success rate of 57%. Based on their experience and feedback from an industry-led survey EMA recommends enhancements in scope and timing of eligibility, flexibility of scientific advice and knowledge building to support the marketing authorisation. An important conclusion is that due to the considerable resource assessment, it is critical to select the drugs that are most likely to benefit.

### Impact of Expedited Development Tools on Overall Development Timelines

In general, it is hard to quantify the overall impact of expedited pathways on the development timelines for pharmaceuticals as many other factors are involved. Several authors have attempted such analyses, but the results are difficult to interpret.

A study from 2009 [19] evaluated 51 new medical entities for oncology indications that had been approved by FDA in the period 1995 (upon introduction of Accelerated Approval) to 2008 (prior to the availability of BTD). This included 19 (37%) drugs with Accelerated Approval designation and 32 (63%) drugs that underwent regular approval. The median development time for both pathways of drugs was essentially the same (7.3 versus 7.2 years). Shea et al. focussed on BTD and compared cancer drugs with and without BTD for 29 drugs approved by FDA between 2013 and 2015 [20]. Drugs with BTD (12/29; 41%) reached the market faster than those without (17/29; 59%). This was mainly due to reduced development timelines as median time from submission of an investigational new drug application (IND) to submission of a new drug application (NDA) or biologics license application (BLA) was 2.2 years shorter. In addition, more drugs with BTD (8/12, 67%) were approved based on data from Phase 1 or Phase 2 trials only (compared to 4/17, 24% for non BTD drugs), as also shown by wide range in the development time for new oncology drugs of 4.0–9.6 years [19–21].

Liberti et al. found that the impact on overall development times (calculated as time from IND date to dossier submission) of drugs using any expedited pathway in the US depends on the exact routing that is followed [21]. They reported the shortest median development time of 1458 days for those drugs with accelerated approval, priority review and breakthrough designation. Products that benefited from fast-track designation or priority review alone had the longest development timelines (median of 2620 and 3515 days, which is longer than the median 2148 days for drugs not benefiting from any expedited pathway. The authors suggest that it would be worthwhile to explore the underlying factors that influence development. Such knowledge could also allow to better predict the impact of the expedited pathways and could play a role in rationalising their use and economise resources.

### Impact on Review and Approval Timelines

Studies that analysed the impact of Priority Review on marketing application review times [21, 22] concluded that median review time for drugs using priority review or other expedited pathways (in US and Canada over various time periods) was substantially reduced. Combinations of more than one expedited pathways reduced review times by more than half [21]. The fastest combinations were Fast Track Designation + Accelerated Approval + Priority Review + BTD, with a median approval time of 145 days and Accelerated Approval + Priority Review + BTD, with a median approval time of 166 days (compared to 242 days and 365 days for drugs with priority review alone and standard review respectively). For drugs using any of these pathways the acceleration was on average four months (median of 243 days compared to 365 days standard review). From this perspective it is interesting to note that Wang et al. [23] showed that between 2011 and 2020 only 14 out of 410 new medical entities approved by FDA were granted all four expedited pathway designations and that 12 of them were oncology drugs.

Similarly, an analysis of all centrally authorised drugs in the EU revealed that the mean time for drugs reviewed under Accelerated Assessment was 248 days compared to 431 days for standard assessment in the EU during the 5-year period 2016–2020 (reduction of review time by 42%) [15]. The 5-year analysis of PRIME concluded that PRIME had a positive impact, reducing overall time to marketing authorisation, mainly due to a shorter clock-stop duration [16].

An important observation from above analyses is that “combined benefits” have been shown to have the largest impact on both development and review timelines. However, even though applying different expedited pathways simultaneously is quite frequently seen, this is rarely officially offered by the agencies. The only official procedure is pioneering drug designation (Sakigake) in Japan, providing close Agency interactions and accelerated approval timelines. In China, candidates for BTD are also eligible for priority review and possible rolling submission. Agency interaction and accelerated approval timelines are also foreseen for PRIME (which is essentially built as a procedure using close interaction for specific drugs to allow for accelerated assessment), but for drugs under PRIME, eligibility for accelerated assessment still needs to be confirmed.

The EU adaptive pathway approach, which ran as a pilot project under the Innovative Medicines Initiative between 2014 and 2018, provided such an end-to-end approach but is no longer pursued. During multi-stakeholder activities many concerns with an adaptive licensing approach were addressed but some gaps regarding pricing, reimbursement, interpretability of real-world evidence and also intellectual property questions remained.

Given the positive impact observed on development and approval timelines, an end-to-end holistic approach across ICH regulators would be the ideal way forward.

### Approvals Based on Based on Early Evidence Data Packages

With approvals based on early evidence (e.g., surrogate endpoints, small or selective groups of patients) data packages, there is a higher amount of uncertainty at the time of approval, which is usually outweighed by the expected benefit to patients but requires post-authorization evidence for confirmation. A particular challenge arises from the scenario that the required post-authorisation studies may not support the benefit/risk balance in the conditionally approved indication at all, or only support benefit/risk in a specific patient subset [24]. In such a scenario, regulators will consider, based on the review of the totality of data available at this timepoint, whether the conditional or accelerated approval has to be revoked and the drug taken off the market, or the period for post-approval data collection should be extended or the indication should be restricted to the population with a positive benefit/risk balance. A prominent example in the EU is the revocation of the conditional marketing authorisation for lartruvo (olaratumab) in combination with doxorubicin for the treatment of patients with soft tissue cancer following review of data from the ANNOUNCE study revealing no survival benefit gain versus doxorubicin monotherapy [25]. In the US, between 1992 and 2021, approximately 165 oncology drugs have received accelerated approval. Of those, 69 have been converted to full approval. A total of 10 approvals were withdrawn because the initially anticipated benefit could not be confirmed. The main reasons were less than expected efficacy in the confirmatory trials and/or a changing unmet medical need landscape with efficacious alternative substitutes having become available [26].

The limited validity of the initial conditional marketing authorisation (1–2 years), either by law (Australia, EU, UK and Switzerland) or through planned drafted re-assessment of the authorisation after the confirmatory trials have been finalized (as in US) alongside with an agreed timetable aims to facilitate timely completion of confirmatory clinical studies and keeps pressure on sponsors after approval. In the US, as part of the next 5-year reauthorisation cycle of user fees discussion, drafts are ongoing (not final yet) to amend the accelerated approval pathway to (1) require FDA to agree on the conditions for the required confirmatory study for a drug approved under the accelerated approval pathway by the time the accelerated approval is granted and (2) “streamline” the administrative process for withdrawing an accelerated approval when the sponsor fails to perform due diligence in completing the confirmatory study or when the post approval trial has failed to confirm clinical benefit.

### Potential Impact on Sequence of Submissions

The increasing availability of expedited pathways across the world is likely to have an impact on the regulatory submission sequence and launch strategy of drug developers. In future, first wave submissions might no longer start with US/EU but utilize the option of collaborative review such as Orbis/ Access as first wave. Considering the ongoing rapid rehaul of the Chinese regulatory system and depending on commercial considerations, China might in the future be considered as first wave country.

Although the availability of new, expedited regulatory pathways is welcomed, there are also challenges. An important challenge is that not all pathways are equally accessible and used. For example, in China the program is not open for drugs approved already outside China and developers face difficulties in acceptance of clinical data generated outside China. To maintain Japanese pioneering drug designation (Sakigake), first submission must occur in Japan or in parallel to another country. This could be overcome by involving China and Japan in clinical trials earlier in development. The implementation of the ICH guideline E17 on general principles for planning and design of multi-regional clinical trials is crucial in all ICH countries including China to increase the acceptance of applications with foreign clinical data. The Japanese pioneering drug designation (Sakigake), although well-valued, is very much Japan focussed and lacks the global outlook. In general, regional differences in scope and requirements will become a more important aspect of the global submission strategies.

### International Collaborations

Forward-looking, in addition to further expanding on existing expedited regulatory pathways, regulatory agencies are looking increasingly toward collaboration pathways to share expertise and optimize resources. These pathways rely on work-sharing, collaborative review, exchange of expertise and/or shared assessment aids and technology. Currently active examples are Orbis, Access and ASEAN JACG and most recently the EU OPEN initiative (thus far restricted tor COVID vaccines and therapeutics). These initiatives are expanding and maturing as more countries join and experience increases.

For regulatory agencies, advantages include efficient use of resources through work-sharing, mutual learning and the possibility to strengthen expertise in a certain area as well as increase of global alignment, while maintaining independence in decision-making. Participation in these programs by pharmaceutical companies necessitates early planning of the sequence of submission, frequently frontloading of work and taking into account the regulatory and scientific guidelines of the major markets. Although this initially may complicate development (working with many different, sometimes contradictory guidelines, short response timelines), increasing collaboration between regulators may also trigger further harmonisation in regulatory requirements. Furthermore, the submitted dossiers will be increasingly similar and ideally, a consolidated list of questions will facilitate a smoother response process for developers.

## Conclusions

Over the past years, a tendency has been seen for ICH members and beyond to modernize their regulatory systems to implement different expedited regulatory tools to ensure faster development and approval of innovative drugs in areas of unmet medical need. This has already resulted in new regulatory paradigms in major markets like China (providing BTD, priority review and conditional approval) and Brazil (now accepting less than comprehensive dossiers for rare diseases or diseases with unmet medical needs). MHRA by participating in the Project Orbis and Access Consortium as well as by establishing other innovative regulatory tools is able to approve certain medicines ahead of the European Union.

Despite the differences seen in all expedited approval pathways in ICH member states and regions they share a common denominator and are foreseen for drugs addressing an unmet medical need.

It is expected that additional regulatory acceleration options will arise in the near future, by further increasing collaborative work-sharing such as already implemented in the Access consortium and Orbis, building on existing pathways and revision of existing tools. In addition, new knowledge in science, new approaches to regulatory review taking advantage of digitalization such as dynamic regulatory assessment [27] may facilitate further reduction of regulatory review timelines while maintaining a scientifically robust regulatory assessment.

The changing regulatory environment will require sponsors to rethink drug development and regulatory strategy and may cause a transformative disruption to the current sequence of regulatory submissions, favouring those countries/collaborations that are best equipped to make innovative drugs for areas of unmet medical need available to patients in the shortest time frame.

## Data Availability

All data and datasets analysed and described in this article are publicly available.
